# EGF-receptor phosphorylation and downstream signaling are activated by genistein during subacute liver damage

**DOI:** 10.1007/s10735-023-10127-8

**Published:** 2023-05-25

**Authors:** Erick Ayala-Calvillo, Lourdes Rodríguez-Fragoso, Elizabeth Álvarez-Ayala, Alfonso Leija-Salas

**Affiliations:** 1 Facultad de Farmacia, Universidad Autónoma del Estado de Morelos. Av Universidad, 1001 Col. Chamilpa CP 62210, Cuernavaca, Morelos México; 2 Centro de Ciencias Genómicas, Universidad Nacional Autónoma de México, Av. Universidad 2001, CP62210 Col. Chamilpa, Morelos Cuernavaca, Mexico

**Keywords:** Genistein, EGFR signaling pathways, Liver damage.

## Abstract

The epidermal growth factor receptor (EGFR) plays an important role on hepatic protection in acute and chronic liver injury. The aim of this study was to investigate the role of genistein on EGFR expression, phosphorylation and signaling pathways in experimental subacute liver damage induced by carbon tetrachloride (CCl_4_). We used male Wistar rats that were randomly divided into four groups: (1) Control; (2) Genistein 5 mg/kg per oral; (3) Subacute liver damage induced by CCl_4_ 4 mg/kg subcutaneously; and (4) Animals received CCl_4_ and genistein at the dosage indicated. The effect of genistein on EGFR expression, phosphorylation and signaling pathways were investigated by western blot and densitometric analyses. Histological changes were evaluated on slices stained with Hematoxylin-Eosin and Masson´s trichromic, as well as an immunohistochemical analysis for proliferating cell nuclear antigen (PCNA). Additionally, pro-inflammatory cytokines and liver enzymes were quantified. Our study showed that genistein increased EGFR expression, EGFR-specific tyrosine residues phosphorylation (pY1068-EGFR and pY84-EGFR), signal transducer and activator of transcription phosphorylation (pSTAT5), protein kinase B phosphorylation (pAKT) and PCNA in animals with CCl_4_-induced subacute liver damage. It was found a significant reduction of pro-inflammatory cytokines in serum from animals with subacute liver damage treated with genistein. Those effects were reflected in an improvement in the architecture and liver function. In conclusion, genistein can induce a transactivation of EGFR leading to downstream cell signaling pathways as early events associated with regeneration and hepatoprotection following subacute liver damage.

## Introduction

The chemically-induced hepatotoxicity is followed by compensatory liver regeneration, which involves an interplay of several signaling pathways (Michalopoulos 2007). The EGFR signaling system plays a pivotal role for liver regeneration and maintaining liver homeostasis (Carver et al. 2002; Paranjpe et al. 2010; Tsagianni et al. 2018). EGFR is a thyrokine kinase-like receptor that is activated by phosphorylation of specific residues after ligand stimulation. The phosphorylated tyrosine can activate downstream signaling pathways are the AKT/phosphoinositide 3-kinase (PI3K), the phospholipase C (PLC-γ1), and the signal transducer and activator of transcription (STAT) controlling proliferation, differentiation, and survival (Schlessinger 2002; Jorissen et al. 2003). Therefore, EGFR pathway may be a critical factor associated with the regeneration and hepatoprotective effects (Komposch and Sibilia 2015).

Genistein (4,5,7-trihydroxyisoflavone) is a major isoflavone in soybeans, and it possesses estrogenic and antioxidant activity (Ganai and Farooqi 2015; Lecomte et al. 2017). For many years genistein has attracted extensive interest due to its important role in preventing and treating common disorders, such as osteoporosis, cardiovascular diseases, diabetes, and cancer. (Palanisamy et al. 2011; Maulik et al. 2012; Messina 2014; Tuli et al. 2019). Previous studies have demonstrated that genistein is an inhibitor of protein-tyrosine kinases.

attenuating growth factor and cytokine, as EGFR ligands (Liu et al. 2002; Kim et al. 2011). Previously, this group have reported that genistein is able to modify liver fibrosis and improves liver function by inhibit EGFR expression in rats with fibrosis induced by chronic administration of carbon tetrachloride (Rodriguez et al. 2016). Interestingly, several research groups have also described the hepatoprotective role of genistein in acute liver damage induced by chemicals (Kuzu et al. 2007; Ali et al. 2015; Semeniuk et al. 2021). Therefore, the aim of the present study was to investigate the role of genistein on EGFR expression, phosphorylation and signaling pathway in a CCl_4_-induced subacute liver injury model of rats. We hypothesized that genistein might modify the expression and phosphorylation of EGFR in subacute liver damage by transactivating this receptor, and therefore it improves the architecture and liver function.

## Materials and methods

### Animals

Male Wistar rats weighing 200 g were obtained from Envigo Laboratories S.A. de C.V., Mexico. The animals were kept at an average ambient temperature of 25 °C in a temperature and with 12-hour light-dark cycle. Animals were fed a standard diet (Standard Purina Chow) and had water *ad libitum*. The study was approved by the Institutional Research Ethics Committee (No. 2689) and was conducted in accordance with the Guide for the Care and Use for Laboratory Animals (National Research Council, 2011).

### Model with subacute liver damage caused by CCl_4_

The rat model was established using the method described previously (Kuzu et al. 2007). In order to induce subacute liver toxicity, twenty-four Wistar rats were randomly divided into four groups (six rats/group). Group 1 (control), animals received the vehicle only (PBS). Group 2 (Genistein), animals were treated with 5 mg/kg of body weight of genistein per oral, daily for 4 weeks. Group 3 (Subacute liver damage), animals were treated with 4 mg/kg of body weight of CCl_4_ in mineral oil subcutaneously three times per week during four weeks (subacute damage). Group 4 (Liver damage + genistein), animals received CCl_4_ and genistein at the dosage indicated. Genistein and CCL_4_ were obtained from Sigma Chemical Co., St. Louis, MO. The genistein dose was chosen in accordance with previous results carried out in animals treated with this drug (Semeniuk et al. 2021). After the treatments, animals were deprived of food, but not of water, for 12 h, and were sacrificed with pentobarbital sodium (150 mg/kg of body weight), i.p.). The rats blood samples were centrifuged to separate serum, which was kept at – 70 °C until analysis. A portion of the liver was removed for histopathological analysis, the remaining liver was cut in pieces and rapidly frozen to – 70 °C for extraction of hepatic proteins. cuts. cuts. These experimental procedures were performed in accordance with the guidelines for animal experimentation of the Autonomous University of the State of Morelos (Mexico), Welfare Act Public Laws of Morelos State (Mexico) and reported according to the ARRIVE guidelines (Lilley, et al. 2020).

### Western blotting

Liver extracts were prepared from pieces of liver tissues from every rat using lysis buffer (20mM HEPES, 2 mM EGTA, 50 mM β-glycerol phosphate, 5 mM sodium fluoride, 50 mM dithiothreitol, 100 mM phenylmethylsulfonyl fluoride, 1% Triton X-100, 10% glycerol) and phosphatase inhibitor cocktails (Roche, Diagnostics, Indianapolis, IN) and homogenized. Lysates were centrifuged 17,005 x g during 10 min. The supernatant was collected, and the total protein content was quantified with the Pierce BCA Protein Assay kit (Thermo Fisher Scientific). The cell lysates were stored in − 70 °C until use, and the proteins were separated by SDS/PAGE in 8% polyacrylamide gel at 100 V for 1.5 h, and transferred to nitrocellulose membrane (Bio-Rad, Hercules, CA). The blots were washed three times in Tween-Tris-buffered saline (TTBS; 50 mM Tris/HCl, pH 7.5, 0.5% Tween-20, 0.15 M NaCl), blocked for 45 min at room temperature with 5% non-fat milk in TTBS and incubated overnight at 4 °C with the primary antibody. After washing with TTBS, the blots were incubated with anti-rabbit or anti-mouse secondary antibody conjugated with horseradish peroxidase (HRP) for 1.5 h at room temperature. The antibodies used in the experiments were as follows: EGFR (1:500, #4267, Cell Signaling Technology, Danvers, MA, USA), phospho-EGFR Y845 (1:500, #2231, Cell Signaling), phospho-EGFR Y992 (1:500, #2235, Cell Signaling), phospho-EGFR Y1068 (1:500, #2236, Cell Signaling), AKT (1:500, #9272, Cell Signaling), phospho-AKT (1:500, #9271, Cell Signaling), STAT5 (1:500, #9363, Cell Signaling), phosphor-STAT5 (1:500, #9351, Cell Signaling), PLC-γ1 (1:500, sc-7290, Santa Cruz Biotechnology, CA, USA), phospho-PLC-γ1 (1:500, sc-136,186, Santa Cruz Biotechnology), β-actin (1:500, sc-47,778 S, anta Cruz Biotechnology), secondary antibody anti-rabbit HRP (1:2000, #7074, Cell signaling, Technology Inc.) and secondary antibody anti-mouse HRP (1:1500, A2304, Sigma-Aldrich). They were then washed in TTBS and the bands were visualized using SuperSignal West Pico Chemiluminescent Substrate (Thermo Scientific, Rockford, IL, USA). The intensity of each band was imaged and quantified by QuantityOne® software (Bio-Rad, USA).

### Immunostaining for PCNA

Liver sections were cut at 4 m, mounted on slides coated with 3 aminopropyltriethoxysilane (Sigma-Aldrich Co.; MO, USA), air-dried at room temperature, and heated at 60 °C on a hot plate for a few seconds until the paraffin melted. After deparaffinization, the slides were incubated in 2% hydrogen, rehydrated in 95% ethanol, and rinsed again in PBS. When incubation was performed, the sections in 2 N HCl were incubated at room temperature for 30 min and subsequently washed three times in PBS. Samples were incubated for 45 min with monoclonal anti-PCNA 1:200 dilution (1:200, sc-56, Santa Cruz Biotechnology, CA, USA) for 30 min at RT. The liver pieces were then incubated with a biotinylated rabbit anti-mouse (dilution 1:200 in PBS) with 1% bovine serum albumin at 42 °C for 20 min. After a time, they were incubated with avidin DH-biotinylated horseradish peroxidase H complex (Vectastain Elite ABC Kit; Vector Laboratories, CA, USA) for 20 min at RT. Finally, the slices were exposed with 0.05% diaminobenzidine tetrahydrochloride and 0.01% hydrogen peroxide substrate solution in 0.05 M Tris-HC1 (pH 7.6) for 3 min and then washed. As positive control tissue, we used a section of regenerating rat liver. The image was analyzed using an Image-Pro® Imaging Software (Media Cybernetics Inc.). To get average positive level of each sample, eight microscopic fields of 100× magnification were selected which included three characteristic fields each of significant, medium and a few positive cells. PCNA labeling index is the percentage of immunohistochemical staining positive cells in 1000 cells counted.

### Analysis of pro-inflammatory cytokines

The serum level TNF-α, IL-1β, IL-12p70, IL-2, RANTES and VEGF were determined using a commercially available enzyme linked immunosorbent assay (ELISA) Kit (Biosource International Inc., Camarillo, CA, USA) according to the manufacturer’s instructions. Briefly, a 96-well micro well plate, designed for ELISA (Fisher Scientific) were coated with TNF-α, IL-1β, IL-12p70, IL-2, RANTES and VEGF capture antibody and incubated overnight at 4 °C. After incubation, the capture antibody was removed by washing the plate three times with wash buffer (PBS and 0.05% Tween-20). Assay diluent (PBS and bovine calf serum) was added to each well (blocking non-specific binding) and incubated at room temperature for 1 h. Supernatants from the treated cells and controls (appropriately diluted), and TNF-α, IL-1β, IL-12p70, IL-2, RANTES or VEGF standards, were added to the wells and incubated for 2 h. After incubation with samples and standards, detection antibody was added for 1 h and this was followed by addition of substrate. Substrate was converted to a colored product that was measured at 450 nm on a Multimode Plate Reader Victor X3 (Perkin Elmer, USA) at 405 nm.

### Histopathological examination

Liver tissue samples were fixed in 4% formalin solution during 48 h and transferred in a cassette and immersed in paraffin a 60 °C. Samples were processed and slices of 2 μm were cut using a microtome Microm (Carl Seis). Liver samples were stained with Hematoxylin-Eosin and Masson´s trichromic. Histopathological examination was performed by a pathologist who specialized in this field. Tissue sections were reviewed independently for two pathologists. Overall interobserver difference was 6%. In case of differing results consensus was reached by joint the analysis. Fibrosis grading and staging was performed based on METAVIR score, where: F0 corresponded to no fibrosis; F1 corresponded to fibrous portal expansion; F2 corresponded to few bridges or septa; F3 corresponded to numerous bridges or septa; and F4 corresponded to numerous bridges with regeneration nodules (cirrhosis).

### Liver function analysis

The blood collected from the rats was centrifuged to separate plasma, which was kept at − 70 °C until analysis. Alanine aminotransferase (ALT), aspartate aminotransferase (AST) and alkaline phosphatase (ALP) enzyme activity were measured with commercial kit (ELITech, Mexico) in Olympus AU 600 autoanalyzer. Liver enzymes were quantified by colorimetry using a commercial reagent kit (ELITech, Mexico), values were expressed as international units per liter (U/L).

### Statistical analysis

The statistical analysis was performed using SPSS 17 Real Stat software (Armonk, NY, USA). The statistical differences between groups were determined by ANOVA, followed by Tukey’s test. A p < 0.05 values were considered as statistically significant.

## Results

### Expression and phosphorylation of EGFR

**Fig. 1:**
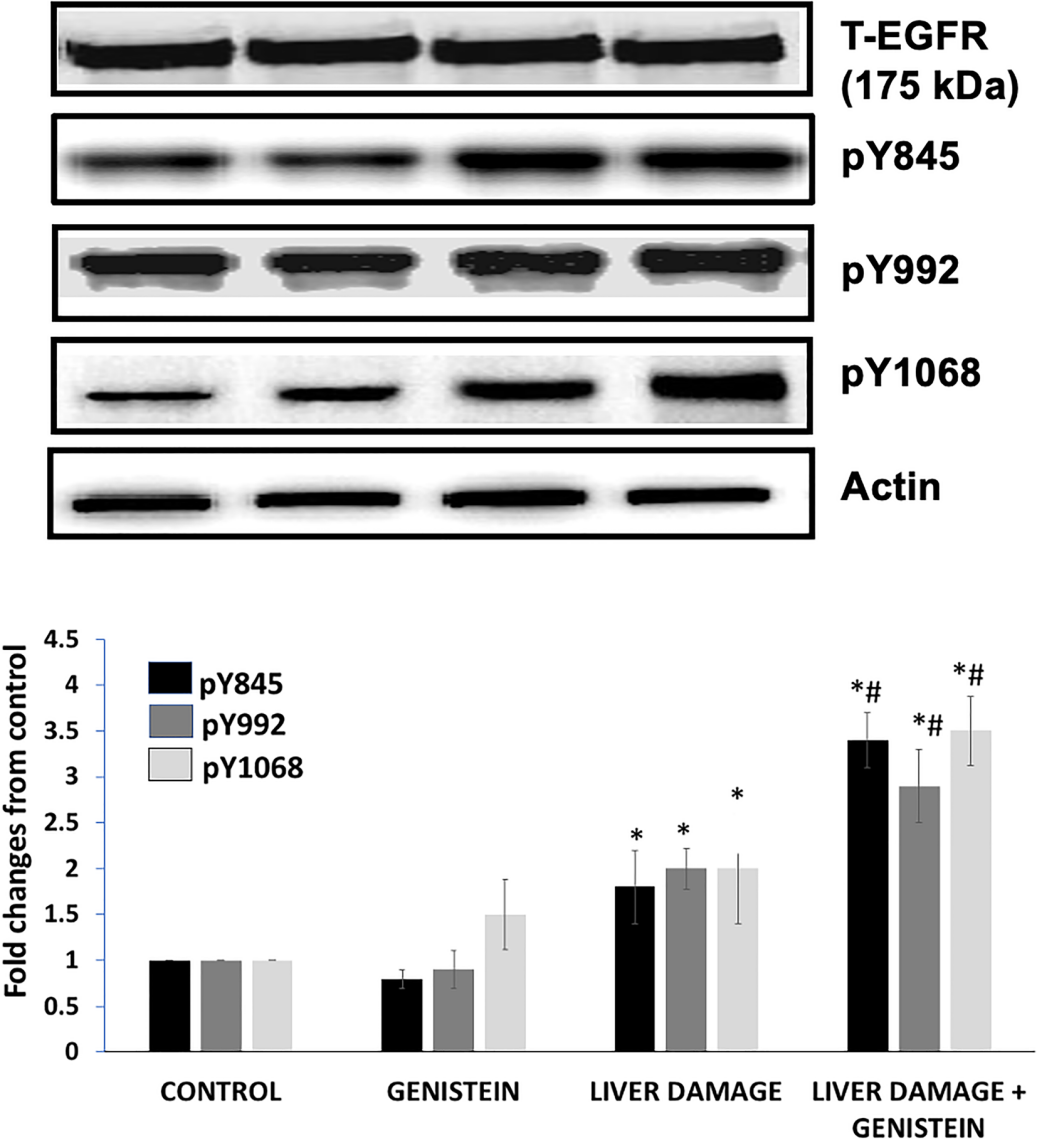
Effect of genistein on expression and phosphorylation of EGFR during experimental CCl_4_-induced subacute liver damage. An increase in total protein and phosphorylation from livers with CCl_4_-induced subacute liver damage was observed. Genistein significantly increased total protein and pY845 and pY1068 EGFR. The analysis was determined by Western blot a semiquantitative analysis of the expression levels of p‑EGFR (Y845, Y992 and Y1068), β-actin was used as a loading control. Bars show the mean values ± standard deviations of the band density normalized to the total protein. n = 8, *p < 0.05 as compared to control group; #p < 0.05 as compared to liver damage group, respectively using an ANOVA and Tukey’s test

In order to know whether both hepatoprotective effects of genistein were also associated with signs of hepatic regeneration, we performed the analysis of EGFR expressions and phosphorylation by western blot and densitometry analyses. (Fig. 1). Significant changes were observed in EGFR-specific tyrosine phosphorylation residues using a Western blot (Fig. 1). Densitometric analysis indicated that genistein induced EGFR tyrosine phosphorylation in the liver during subacute damage. A significant increase in phosphorylation at pY845, pY992 and pY1068 EGFR in group with subacute liver damage treated with genistein, 88%, 45% and 75% respectively, compared with the group of CCl_4_-induced subacute liver damage (p < 0.05). Therefore, these results indicate that genistein induces EGFR tyrosine phosphorylation at specific tyrosine residues during subacute liver damage. It was interested to note that genistein by itself induced pY1068 EGFR expression (50%) as compared with control group.

### Activating complex downstream signaling cascades of EGFR

**Fig. 2:**
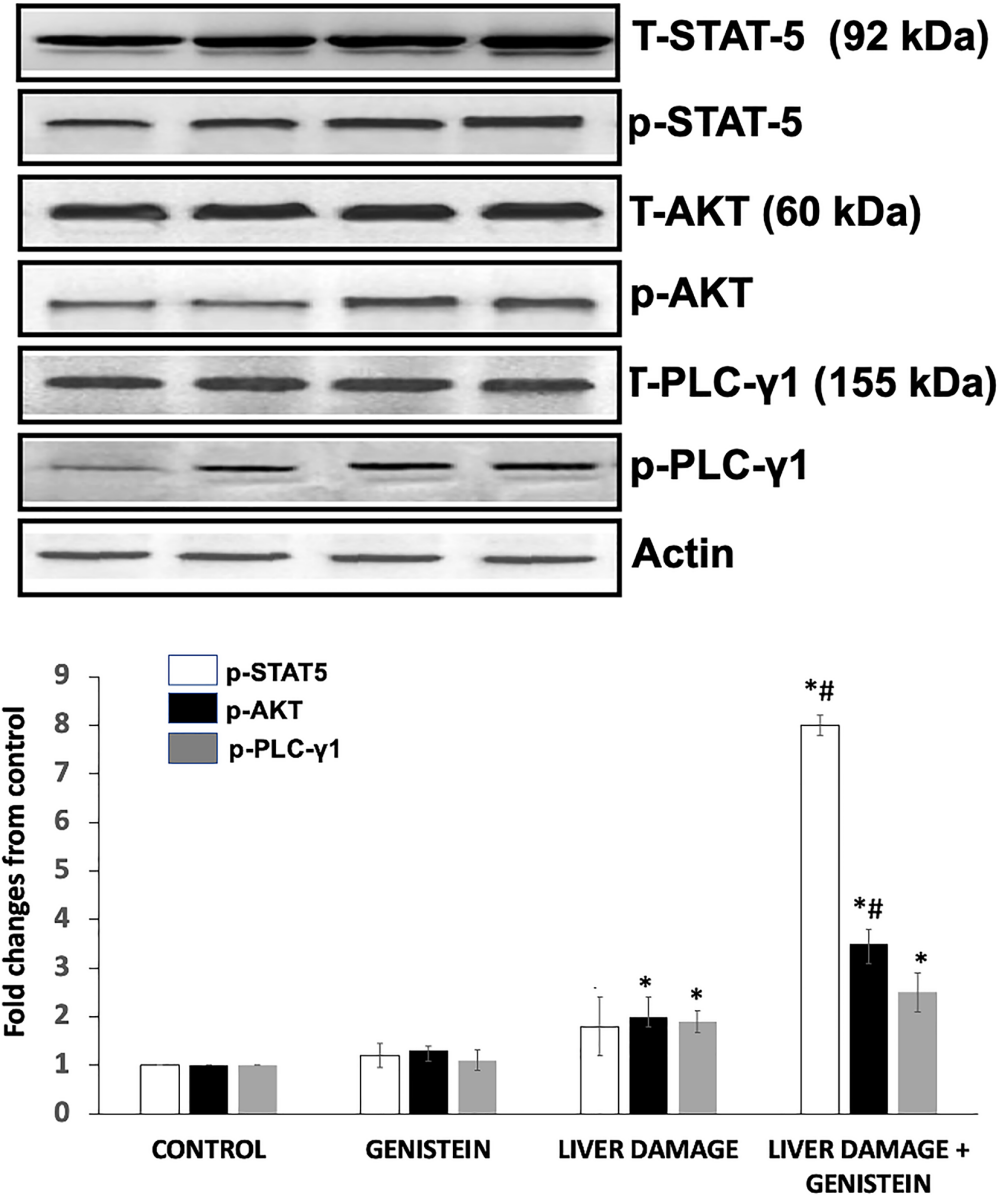
Effect of genistein on the activating complex downstream signaling cascades of EGFR. Western blot and semiquantitative analysis of the expression levels of AKT, p-AKT (S473), PLC-γ1, p-PLC-γ1 (Y1253), STAT5, pSTAT5 (Y694) and β-actin (used as a loading control) in liver tissue were evaluated. Liver from rats treated with CCl_4_ showed an increase in phosphorylation of all proteins. Genistein induces even more pAKT and pSAT5 phosphorylation in animals with liver damage. Bars show the mean values ± standard deviations of the band density normalized to the total protein. n = 8, *p < 0.05 as compared to control group; #p < 0.05 as compared to liver damage group, respectively using an ANOVA and Tukey’s test

After evaluating EGFR expression and phosphorylation in the liver during subacute damage, we assessed the expression level of the AKT, PLC-γ1 and STAT5, and their phosphorylated counterparts to know the status of proliferative and survival activity after all treatments. The effect of genistein on those proteins after CCl_4_-induced subacute liver damage was evaluated by western blot and densitometry analyses (Fig. 2). STAT-5 proteins normally are involved in a variety of cell signaling pathways. Therefore, we examined the total and phosphorylation status of STAT-5 in all groups. We found that neither the expression of STAT-5 at the total protein levels nor STAT-5 phosphorylation were increased in the liver during subacute damage. However, rats with genistein treatment showed a significant increase in STAT-5 phosphorylation (4.4-fold) in rats with subacute liver damage, as compared with subacute liver damage group (p < 0.05) (Fig. 2). AKT also contributes to cell proliferation via pY1068 EGFR. Therefore, it was interesting to analyze the total and phosphorylation status of AKT in all groups. We found that in the liver during subacute damage did not alter AKT at the total protein level; however, it was observed a slightly increase at AKT phosphorylation as compared with control group (100%) (p < 0.05) (Fig. 2). Animals with subacute liver damage and treated with genistein showed an important increase in both at total protein level and pAKT (75%) (p < 0.05) as compared with animals with subacute liver damage. PLC-γ1 is activated in response to various extracellular stimuli and mediate intracellular signal transduction. Our results showed a slight increase in PLC-γ1 at total protein levels and p PLC-γ1 in rats with subacute liver damage, as compared with control group (90%) (p < 0.05). However, animals with subacute liver damage treated with genistein also showed a slight increase in PLC-γ1 phosphorylation (30%), which not was statistically significant (Fig. 2). Therefore, our results suggest that the activation of STAT5, AKT and PLC-γ1 is mediated by EGFR in the liver during subacute damage.

### Expression of PCNA in liver damage

**Fig. 3:**
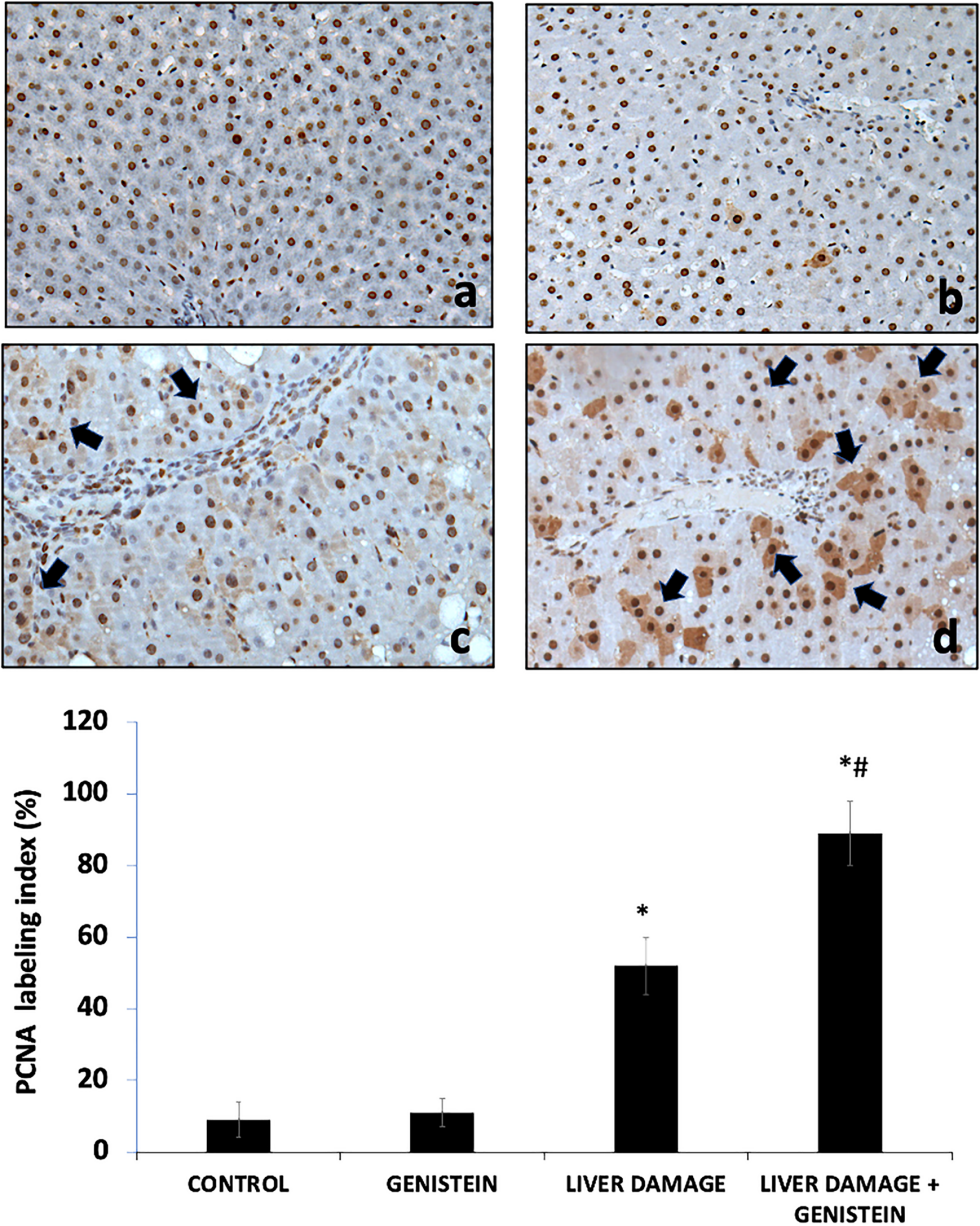
Effect of genistein on PCNA expression and localization in liver. PCNA-positive cells are shown in brown (nuclei) (black arrows). **a** and **b** Liver from the control and genistein groups respectively, scattering positive cells were seen across the parenchyma; **c** PCNA localization in liver sample with CCl_4_-induced subacute liver damage. We can see foci of positive cells across the parenchyma with inflammatory foci. **d** Liver sample of animal with liver damage and treated with genistein. We also observed positive cells in parenchymal. We chose a representative microphotograph of each group, magnification 40×. Bars show the mean values ± standard deviations. *P < 0.05 as compared with control group; n = 8, #p < 0.05 as compared with liver damage group, respectively using an ANOVA and Tukey’s test

We evaluated PCNA expression to establish the status of proliferative activity in the liver during subacute damage (Fig. 3). In the control and genistein groups, showing few hepatocytes with positive nuclei to the PCNA marker. The livers from animals developing subacute liver damage showed a significant increase in PCNA labeling index (6.5-fold) as compared with control group (p < 0.05). This increase indicates the high proliferative activity of hepatic cells after subacute liver damage. In liver sections of animals with liver damage and treated with genistein were also found several positive cells to PCNA; however, the PCNA labeling index was higher than animals with subacute liver damage (70%) (p < 0.05), suggesting an important proliferative activity. Areas where were found positive cells showed characteristics like normal architecture suggesting that necrotic areas were replaced by new hepatic cells.

### Inflammatory signals associated with hepatotoxicity

**Fig. 4:**
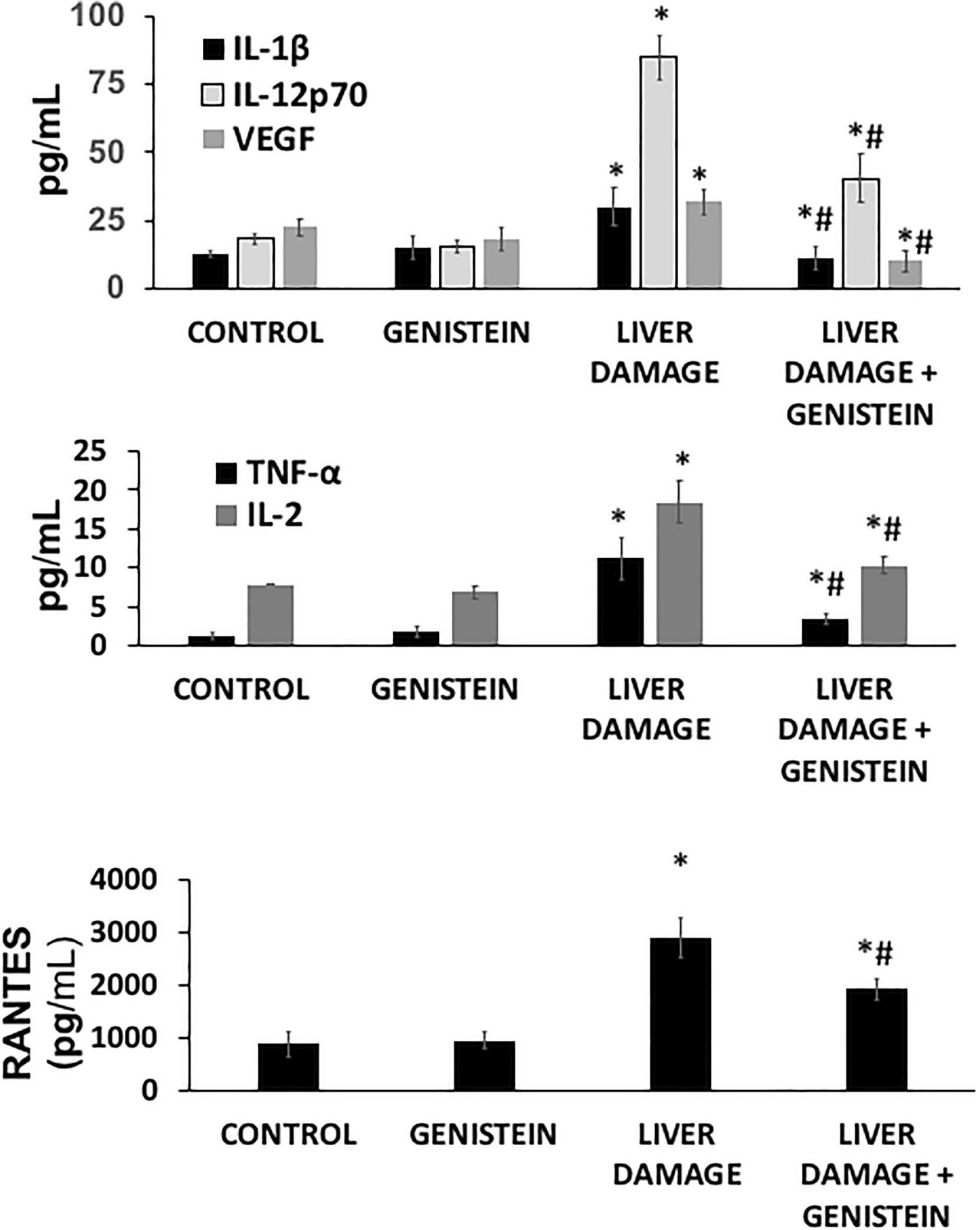
Effect of genistein on pro-inflammatory cytokines levels in rats with CCl_4_-induced subacute liver damage. Cytokines pro-inflammatory were analyzed in serum by an ELISA assay. TNF-α, IL-1β, IL-12p70, IL-2 and RANTES protein levels were found increased in rats with liver damage. Genistein treatment significantly reduced the expression of all pro-inflammatory cytokines in animals with CCl_4_-induced subacute liver damage. Bars show the mean values ± standard deviations. TNF-α, tumor necrosis factor-alfa; IL-1β, interleukin-1 beta; IL-12p70, interleukin-12p70; IL-2, interleukin-2; RANTES, regulated upon Activation/Normal T Cell Expressed and Presumably Secreted; VEGF, vascular endothelial growth factor. n = 8, *p < 0.05 as compared to control group; # p < 0.05 as compared to liver damage group, respectively using an ANOVA and Tukey’s test

To explore the underlying mechanism of genistein on proinflammatory mediators, we detected pro-inflammatory cytokines by ELISA. As shown in Fig. 4, the plasma levels of TNF-α, IL-1β, IL-12p70, IL-2, RANTES, and VEGF were significantly increased in animals with subacute liver damage as compared with control group (11, 2.3, 4.6, 7, 3.2 and 0.4-fold) (all p < 0.05). However, the level of all cytokines was found significantly reduced in group with liver damage and treated with genistein (70, 62, 52, 45, 33 and 68% respectively) as compared with animals with subacute liver damage (p < 0.05). These results suggest that genistein could produce anti-inflammatory effects that mediated protection from liver damage.

### Histopathological analysis

**Fig. 5:**
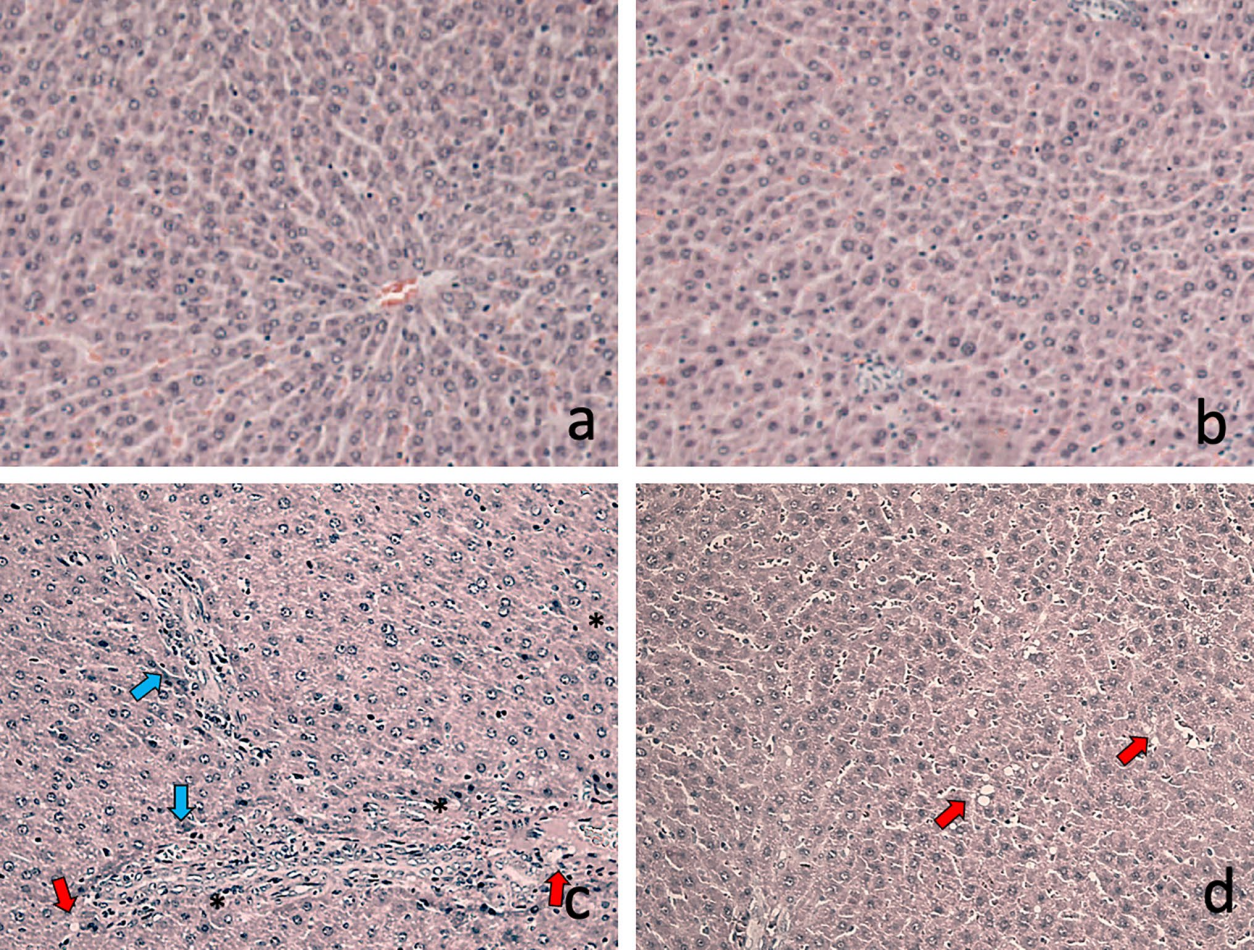
Effect of genistein on liver architecture in rats with CCl_4_-induced subacute liver damage. Representative liver tissue sections from: (**a**) Control rat, and (**b**) Animal treated with genistein, showed normal liver architecture and a normal distribution of collagen (blue); normal distribution and deposition of collagen; (**c**) Animal with CCl_4_-induced subacute liver damage showed presence of inflammatory focus (blue arrow), necrotic zones (asterisk) and steatosis micro and macrovesicular (red arrow); (**d**) Animal with liver damage treated with genistein, liver sections showed an improvement of liver architecture, but macrovesicular steatosis was observed; Liver sections were stained with H&E. A representative microphotograph of each group was chosen, magnification 40×

**Fig. 6:**
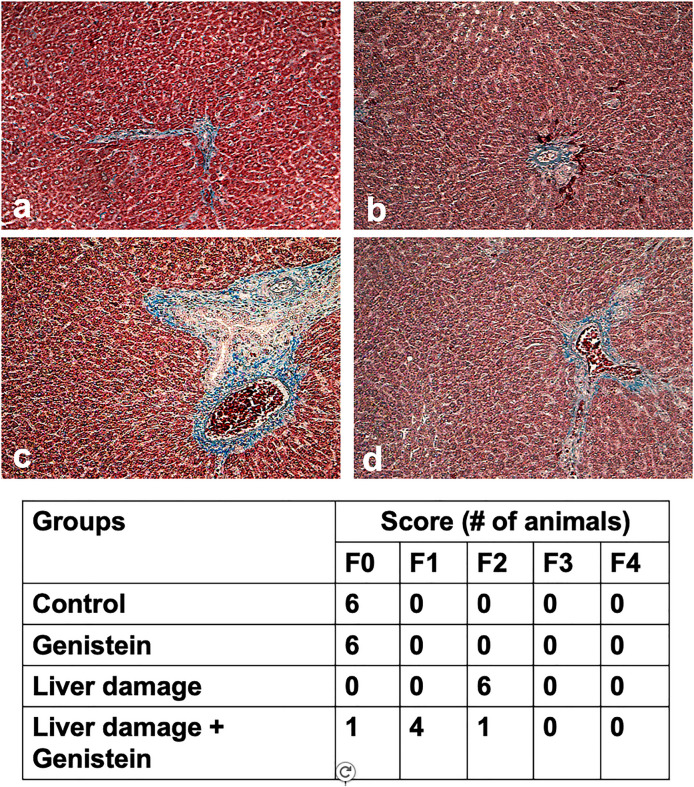
Effect of genistein on collagen deposition. Representative liver tissue sections from: (**a**) Control animal, and (**b**) Animal treated with genistein showed normal architecture and distribution of collagen; (**c**) Animal with CCl_4_-induced subacute liver damage. Animal with subacute liver damage showed extensive collagen deposition surrounded to bile ducts, portal veins, and hepatic arteries; (**d**) Animal with liver damage and treated with genistein showed a liver architecture quite similar than control group. Liver tissue was stained with Masson trichrome; collagen can be recognized by blue staining. F0 corresponded to no fibrosis; F1 corresponded to fibrous portal expansion; F2 corresponded to few bridges or septa; F3 corresponded to numerous bridges or septa; and F4 corresponded to numerous bridges with regeneration nodules (cirrhosis). A representative microphotograph of each group was chosen, magnification 20×

Figure 5 shows the histological findings of representative liver samples from all treatments. Animals with liver damage showed degeneration of hepatocytes, presence of inflammatory infiltrated, steatosis micro and macrovesicular, presence of areas of necrosis with hepatocytes with degenerative changes. However, animals with liver damage and treated with genistein showed a significant improvement in liver architecture; it was observed a reduction of necrotic areas, reduction of inflammatory focus, but small fat drops were observed. Not changes were observed in those animals treated only with genistein. On the other hand, animals with liver damage also showed deposits of extracellular matrix surrounded both blood vessels and bile ducts, which indicated that started the fibrogenesis (Fig. 6). Animals with had the presence of fibrous portal expansion and few bridges or septa. However, genistein reduced the presence of bridges or septa, which suggest that fibrogenesis was stopped.

### Liver function analysis

**Fig. 7:**
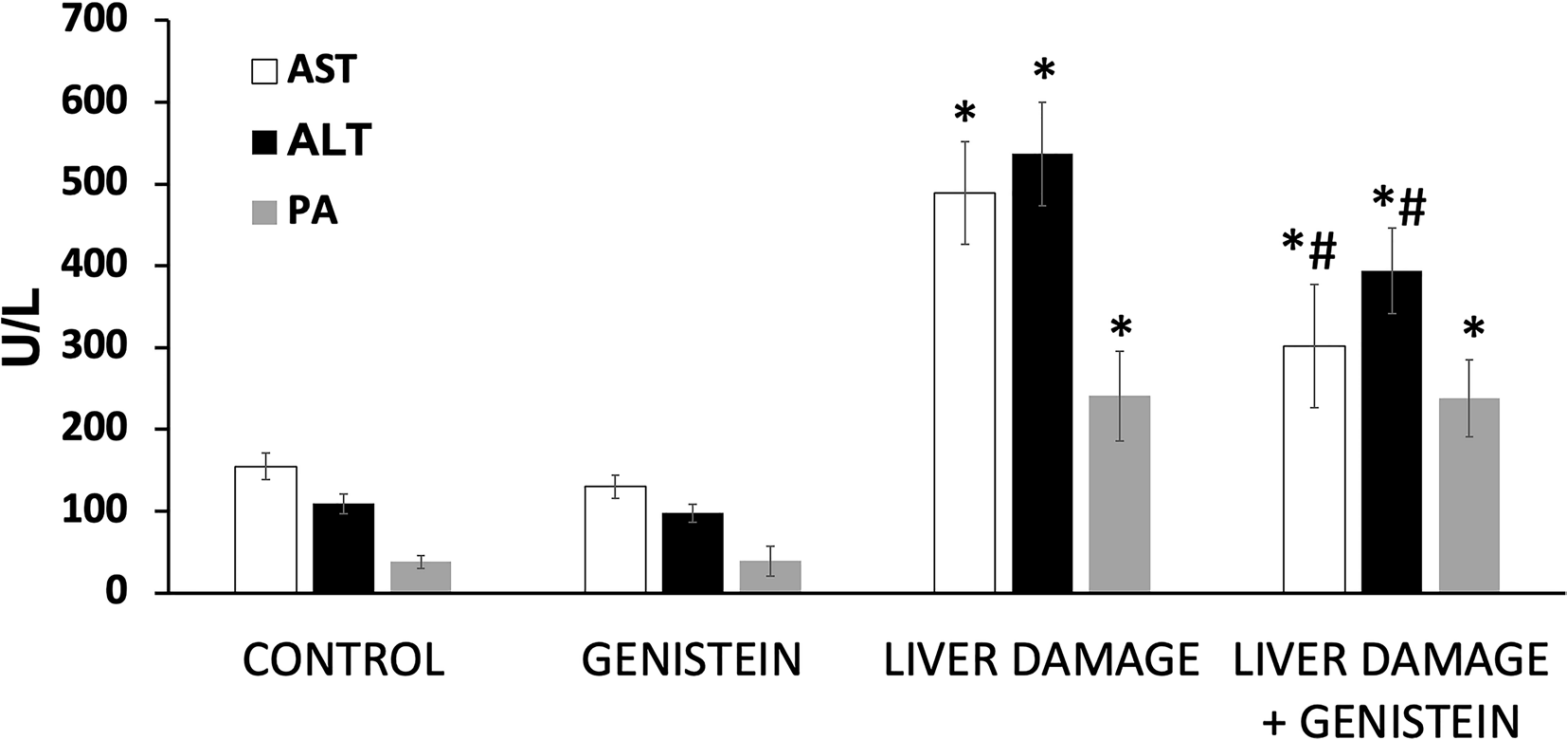
Effect of Genistein on the Activities of Hepatotoxicity markers. The enzymatic activity of alanine aminotransferase (ALT), aspartate aminotransferase (AST) and alkaline phosphatase (AP) was measured in plasma from all groups. Animals with liver injury showed an important increase in liver enzymes. Rats with CCl_4_-induced subacute liver damage treated with genistein showed a decrease in liver enzymes levels. Bars show the mean values ± standard deviations. *P < 0.05 as compared with control group; n = 8, #p < 0.05 as compared with liver damage group, respectively using an ANOVA and Tukey’s test

Significant elevation of AST, ALT and AP levels in the serum usually reflects release of those enzymes from the cytosol of the liver cells when a liver damage occur. To evaluate the extent of liver injury in rats, we carried out an analysis of serum AST, ALT and AP activities. We observed that ALT, AST an AP activities were significantly increased in group with subacute liver damage as compared with control (4.9 fold, 3.1 fold, and 6.3 fold, respectively) (p < 0.05) (Fig. 7), whereas there was a significant reduction of these AST and ALT in animals with liver damage and treated with genistein (60% and 73%, respectively) (p < 0.05). These results suggested that genistein could ameliorate hepatic function in animals with liver injury. As is shown in Fig. 7, oral administration of genistein for three times per week during four weeks in rats did not modify the liver function markers.

## Discussion

Genistein is an isoflavone found in soy products. This phytochemical regulates several biological processes, such as metabolism, inflammation, proliferation, and apoptosis (Sundaram et al. 2019: Lee et al. 2019; Sharifi-Rad et al. 2021; Rasheed et al. 2022). Several researchers have reported the mechanisms of action of this molecule involving numerous targets in living cells, such as the growth factors and their intracellular signaling. (Cui et al. 2017; Chae et al. 2019; Tuli et al. 2019; Garbiec et al.^2022^). In relation to liver disease, previous studies have revealed that genistein inhibits EGFR system and has a protective effect during the processes of acute and chronic liver disease (Rodriguez et al. 2016; Tanjak et al. 2018; Xin et al. 2019; Semeniuk et al. 2021). However, the mechanisms responsible related with the effects of genistein on the expression and phosphorylation of EGFR are not completely understood. Therefore, in present study we used a model of subacute liver damage to study the effects of genistein on EGFR expression, phosphorylation and signaling pathways.

The EGFR is one of the best-studied and characterized receptor tyrosine kinases and plays critical roles during liver development, function, and regeneration (Komposch and Sibilia 2015; Liu et al. 2020, 2022). The EGFR is highly expressed in adult hepatocytes and is activated by several ligands leading to the phosphorylation on multiple tyrosine residues (Ceresa and Peterson 2014). In present work we evaluated the role of genistein on EGFR expression and phosphorylation in animals with subacute liver damage induced with CCl_4_. We found an increased expression and phosphorylation of EGFR in animals, which is consistent with previous reports about the activation of EGFR signaling in toxin-induced hepatotoxicity models (Lanaya et al. 2014; Scheving et al. 2015). It was interesting to note that animals with subacute liver damage and treated with genistein increase even more the EGFR expression and phosphorylation of specific tyrosine residues (pY845-EGFR and pY1068-EGFR). Interestingly, the results of the present study are comparable to other studies, where it was found a remarkable phosphorylation of tyrosine pY1068 in the EGFR in mice and primary human hepatocytes after toxic doses of Acetaminophen (APAP) (Bhushan et al. 2014, 2017). These results suggest that genistein may act also as a receptor tyrosine kinase agonist, rather than a tyrosine kinase inhibitor, after liver injury.

The phosphorylation of specific tyrosine residues of EGFR can serve as attachment sites for various adaptors (e.g., GRB2), cytoplasmic enzymes (e.g., PLC-γ1) or specific factors involved in transcription regulation (e.g., STATs). Together, these signaling effectors and adaptor proteins link activated receptors directly or indirectly to various cell signaling pathways (Kovacs et al. 2015). The increase phosphorylation of tyrosine residues pY845-EGFR and pY1068-EGFR are associated with cell signaling pathways connected with proliferative and survival activity, the main pathways comprise PI-3 K/Akt and STAT ways (Uribe et al. 2021). Our study revealed, important increase in the protein phosphorylation levels of p‑AKT and p-STAT5 in animal with subacute liver damage and treated with genistein. Previous study have shown that genistein-associated cellular proliferation involves the activation of PI3K/Akt pathways in cancer cells. Yang X et al. demonstrated the activation/phosphorylation of Akt and proliferative activity in erbB-2-overexpressing in MCF-7 cells of breast cancers (Yang et al. 2010). Additionally, in HCT 116 colon cells preincubated with genistein derivatives for 24 h before irradiation, the level of both EGFR (pY1068) and pAkt were observed significantly increased (Gruca et al. 2014). Therefore, these results suggested that genistein can be involved in hepatic regeneration after liver injury, by induce specific tyrosine phosphorylation in EGFR. It is well known that when a liver injury occurs loss of hepatocytes triggers events in all hepatic cells that lead to hepatocyte proliferation. Cell proliferation is regulated by EGFR and intracellular signaling pathways during liver regeneration (Shu et al. 2022). According with this results genistein also induces liver regeneration after CCl_4_-induced subacute liver damage.

Various studies have reported that during liver injury several pro-inflammatory cytokines are distinctly associated with the initiation or progression of inflammatory response, oxidative stress, and massive death of hepatocytes (Leverence et al. 2011; Del Campo et al. 2018). In present study, we found that genistein lead to a significant decrease in the production of TNF-α, IL-1β, IL-12p70, IL-2, RANTES and VEGF protein levels in CCl_4_-induced subacute liver damage. Previously has been reported that genistein induce anti-inflammatory effects acting in different cell pathways. For instance, it has been mentioned that genistein may induce an anti-inflammatory effect on macrophages via AMPK activation (Sag et al. 2008). Others have indicated that genistein inhibit inflammation by affecting downstream p38 MAPK signaling (Ge et al. 2020). Similarly, other results revealed that genistein alleviated the pro-inflammatory cytokines such as TNF-α through inhibiting NF-κB activity in LPS-treated RAW264.7 macrophages (Nagaraju et al. 2013). Taken together, these studies suggest that genistein can attenuate inflammatory responses in vitro and in vivo. Our results provide direct evidence for the potential hepatoprotective role of genistein in liver injury by its anti-inflammatory effects.

The induction of liver injury induced with CCl_4_ is one of the most widely used models in the field of hepatology due to spectrum of liver alterations that this hepatotoxin produces (Kuzu et al. 2007; Alqasoumi 2010). In this study, rats exposed during four weeks to CCl_4_ presented significant increase of ALT, AST and AP activities. The histological analysis also revealed the presence of liver inflammation, degeneration, and collagen deposits after CCl_4_-induced subacute liver damage. However, all that pathological findings were ameliorated by effect of genistein. Previous studies have shown that genistein improves several alterations during liver injury as oxidative stress, hepatocyte hypertrophy, and reduces cell death. Even it has been reported that genistein induces changes in lipid deposit and anti-inflammatory modulating miR-451 expression levels mediating a rapid response in acute and chronic hepatic models of inflammation (Gan et al. 2019). The reduction cytokines levels associated with the anti-inflammatory effects correlated with an improvement in hepatic architecture and liver function observed.

In summary, the experimental evidence presented here clearly support the hypothesis that genistein can transactivate EGFR and activate downstream cell signaling subacute liver damage. The pattern of EGFR, STAT5 and AKT phosphorylation found after CCl_4_-induced subacute liver damage might trigger signals to induce cell proliferation and survival leading to liver regeneration. Genistein also reduced pro-inflammatory mediators associated with this process during CCl_4_-induced subacute liver damage. All these effects were reflected in an improvement of architecture and liver function. Genistein has the potential for be used in the treatment of liver disease.

## Data Availability

The datasets generated and/or analyzed during the current study are available from the corresponding author on reasonable request.

## Abbreviations

AKT: Protein kinase B
ALT: Alanine aminotransferase
ALP: Alkaline phosphatase
AST: Aspartate aminotransferase
CCl_4_: Carbon tetrachloride
EGFR: Epidermal growth factor receptor EGFR
ELISA: Enzyme linked immunosorbent assay
IL-1β: Interleukin-1 beta
IL-2: Interleukin-2
IL-12p70: Interleukin-12p70
PCNA: Proliferating cell nuclear antigen
PI3K: Phosphoinositide 3-kinase
PLC-γ1: Phospholipase C
PTKs: Protein-tyrosine kinases
RANTES: Regulated upon Activation/Normal T Cell Expressed and Presumably Secreted
STAT: Signal transducer and activator of transcription
TNF-α: Tumor necrosis factor-alpha
TTBs: Tween-Tris-buffered saline
VEGF: Vascular endothelial growth factor.
